# Bovine Herpes Virus Type 1 (BoHV-1) seroprevalence, risk factor and Bovine Viral Diarrhoea (BVD) co-infection analysis from Ireland

**DOI:** 10.1038/s41598-023-50433-5

**Published:** 2024-01-09

**Authors:** D. Barrett, E. Lane, J. M. Lozano, K. O’Keeffe, A. W. Byrne

**Affiliations:** Department of Agriculture, Food and the Marine, Dublin, Ireland

**Keywords:** Risk factors, Ecological epidemiology

## Abstract

Surveillance of endemic pathogens is essential for disease control, providing an evidence base for policy and advice. Bovine Herpes Virus Type 1 (BoHV-1), the causative agent of Infectious Bovine Rhinotracheitis (IBR), has been found to have high seroprevalence within the Irish cattle population. The aim of the present study was to establish seroprevalence levels for culled cattle in Ireland aged < 30 months and to establish whether BVD exposure and other factors was associated with BoHV-1 exposure. We employed random effects logit models coupled with repeated bootstrap sampling to provide robust estimates. The final dataset contained results for 5273 animals tested over two study years, 2018 and 2020. The animal-level seroprevalence of BoHV-1 was 21.43% (1130/5273; 95%CI: 20.32–22.53%). Univariable analysis suggested that BoHV-1 seropositivity risk was associated with BVDV serodiagnosis status, age, sex, year sampled, herd type, herd-size, and metrics of movement into the herd. Final random-effects multivariable models suggested increased risk associated with increasing herd size of the last herd, movements made by animals during the previous year, and the year the animal was sampled. Despite BVDV status and sex being retained in the final model, repeated bootstrap sampling of the regression model to estimate biased-corrected 95%CI suggested that these associations were not robust. The overall apparent prevalence of BoHV-1 exposure for culled cattle in Ireland declined in 2020 relative to 2018 (from 23.32 to 17.61%). Herd-size and the movement of animals were found to be important factors associated with animal-level risk, but there was less statistical support for sex-based or BVDV status associations.

## Introduction

Bovine Herpes Virus 1 (BoHV-1) infection occurs worldwide (reviewed by^1^, and is an economically significant pathogen due to its impact on production losses^2,3^. BoHV-1 cause life-long infection^4^ that reactivates under stress and may result in viral excretion^5^. Infection with BoHV1 reduces milk yield impacting farm profitability^2,6^.

BoHV-1 is endemic in Ireland^11–13^. Infection with BoHV1 was first described in Ireland in the seventies. Historically, prevalence was low (9% seroprevalence) during the 1980s^14^. Higher prevalence has been described more recently^11,15,16^; with Sayers et al.^7^ reporting 80% of bulk milk samples test positive for BoHV1.

Research has suggested a potential association between BoHV-1 infection and Bovine Viral Diarrhea Virus (BVDV), both in vivo^7,8^; and in vitro^9,10^. High levels of cooccurrence of BoHV-1 and BVDV have been reported in cattle herds in Ireland^7,12,17^. However, the impact of co-infection and the changes in BVDV prevalence over the course of the national BVD Free programme remains unexplored in Ireland.

The Terrestrial manual of the OIE^18^ outlines the requirements for a country to qualify for disease free status; while the Commission delegated Regulation (EU) 2020/689^19^ prohibits vaccination and set sets out the requirement to achieve 99.8% of bovine establishments, representing at least 99.9% of all cattle are free from BoHV-1. Eradication programmes have been successful elsewhere in Europe^1^, and the establishment of a control programme is being considered in Ireland. It is well known animal diseases can reduce efficiency and productive outputs, and therefore requiring increased inputs to overcome disease relative impacts driving up carbon emissions^20^. Control of IBR has the potential to increase farm efficiencies and help mitigate some carbon outputs^21^. As a consequence, the Climate Action Plan 2021^22^ set out the objective of launching an IBR control programme in Ireland.

The primary aim of this study was to establish seroprevalence levels of BoHV-1 infection among younger cattle slaughtered in Ireland. Secondly, the study sought to establish whether BVD exposure, and other factors, were associated with BoHV-1 infection, as part of a broader aim of informing policy and control options.

## Methods

### Sampling and laboratory methods

Information on the survey methods have been published elsewhere^23^, but briefly, a random sample of Irish cattle aged under 30 months was undertaken, using sera collected for routine serological surveillance purposes. For the present study, IBR testing was undertaken during the years 2018 and 2020. No sampling of live animals was undertaken. Blood sampling was undertaken at slaughter i.e. at post-mortem by veterinary professionals and support staff. It should be noted that the animals were not slaughtered for the purposes of this study. Sampled animals, given their age, were reflective of a population managed for BVD risk as all animals were born since the national BVD eradication scheme was initiated (in 2013). A sample size estimation for seroprevalence was undertaken prior to sampling, indicating that a minimum sample size of 1013 per survey (assuming a design prevalence of 10%; SE: 95%; SP: 99%; confidence: 95%; precision: 0.02) was required^24^. Note, all serum was sampled as part of routine national disease surveillance; all samples were collected at post-mortem after routine slaughter at abattoir, and therefore sampling was not subject to animal welfare guidelines.

All testing (both for BoHV-1 and BVDV) occurred within a single laboratory (Cork Regional Veterinary Laboratory), and therefore there was no inter-lab variation. Samples were tested for BVD using the IDEXX BVDV/MD/BVD p80 Protein Antibody Kits, according to the manufacturers’ instructions. According to the manufacturers, the BVD assay has a diagnostic sensitivity (dSE) and specificity (dSP) of 98% and 97%, respectively. Samples were tested for BoHV-1 also using IDEXX gE ELISA test kits, according to the manufacturers’ instructions. The dSE and dSP for this assay has been estimated at 99% and 99.7%, respectively^25^.

### Statistical methods

The outcome variable of interest throughout was the IBR serology test status, which was modelled as a binary outcome. All inconclusive results were considered test negatives throughout. The apparent and estimated true seroprevalence was reported. True seroprevalence was based on the Rogan-Gladen estimator, assuming dSE and dSP reported above, and confidence interval estimated via the Blaker methodology^26–28^.

Generalised linear mixed models were used to model the data, with a random effect included for herd due to the non-independence of multiple observations (i.e. animals sampled) from the same herd. As animals could be clustered via their birth herd or the [last] herd they resided when sampled, two comparative null models without fixed affects were compared. Models with a lower Akaike’s Information Criterion (AIC) and Schwarz Bayesian Information Criterion (BIC) was used as an indicator of the preferred random effect structure.

Descriptive exploration of the independent variables and their relationship with the outcome variable was undertaken. Independent variables included age, breed, sex, movement metrics and herd type (dairy; beef finishing; suckler (beef production where calves are left to suckle from their mothers); other (including mixed enterprises)). Descriptive information on these variables are presented in Table 1.

**Table 1:** Descriptive statistics and univariable associations between selected animal- and herd-level factors and IBR seropositivity risk.

BVD	IBR-	IBR+	Total	OR	P	Upper 95%CI	Lower 95%CI
Neg	3975	1052	5027	Ref			
%	79.07	20.93	100				
Pos	168	78	246	1.928	0.014	1.140	3.259
%	68.29	31.71	100				
Year							
2018	1802	619	2421	Ref			
%	74.43	25.57	100				
2020	2341	511	2852	0.521	< 0.001	0.394	0.688
%	82.08	17.92	100				
Age (q)							
Q1	1009	294	1303	Ref			
%	77	22.56	100				
Q2	975	345	1320	1.537	0.009	1.116	2.118
%	73.86	26.14	100				
Q3	1090	240	1330	0.811	0.226	0.579	1.138
%	81.95	18.05	100				
Q4	1069	251	1320	0.905	0.575	0.638	1.284
%	80.98	19.02	100				
Breed							
AA	720	214	934	Ref			
%	77.09	22.91	100				
CH	752	192	944	0.717	0.117	0.473	1.087
%	79.66	20.34	100				
FR	830	217	1,047	0.756	0.183	0.500	1.142
%	79.27	20.73	100				
HE	608	184	792	0.851	0.462	0.554	1.308
%	76.77	23.23	100				
LM	785	208	993	0.590	0.011	0.393	0.886
%	79.05	20.95	100				
Other	448	115	563	0.663	0.083	0.416	1.056
%	79.57	20.43	100				
Sex							
F	1742	454	2196	Ref			
%	79.33	20.67	100				
M	2401	676	3077	1.386	0.017	1.059	1.815
%	78.03	21.97	100				
Herd change							
No change	1213	247	1460				
%	83.08	16.92	100				
Change	2930	883	3813	1.924	< 0.001	1.413	2.619
%	76.84	23.16	100				
Movements							
0	1189	242	1431	Ref			
%	83.09	16.91	100				
1	1619	437	2056	1.577	0.007	1.133	2.196
%	78.75	21.25	100				
2	984	317	1301	2.095	< 0.001	1.462	3.001
%	75.63	24.37	100				
≥ 3	351	134	485	2.950	< 0.001	1.875	4.642
%	72.37	27.63	100				
Type of last herd							
BEEF	1438	473	1911	1.731	0.001	1.247	2.403
%	75.25	24.75	100				
DAIRY	769	203	972	1.166	0.460	0.776	1.752
%	79.12	20.88	100				
OTHER	376	83	459	1.001	0.996	0.594	1.688
%	81.92	18.08	100				
SUCKLER	1560	371	1931	Ref			
%	80.79	19.21	100				
Type of birth herd							
BEEF	228	58	286	0.832	0.497	0.490	1.414
%	79.72	20.28	100				
DAIRY	2248	660	2908	1.423	0.011	1.083	1.870
%	77.3	22.7	100				
OTHER	184	46	230	0.902	0.731	0.501	1.624
%	80	20	100				
SUCKLER	1483	366	1849	Ref			
%	80.21	19.79	100				
Birth herd size (median: 130; mean: 170.7)							
Q1 (< 71)	1042	258	1300	Ref			
%	80.15	19.85	100				
Q2 (71–138)	1041	279	1320	1.199	0.280	0.863	1.666
%	78.86	21.14	100				
Q3 (139–243)	1044	289	1333	1.385	0.058	0.989	1.940
%	78.32	21.68	100				
Q4 (244–1920)	1016	304	1320	1.474	0.031	1.036	2.099
%	76.97	23.03	100				
Last herd size (Median: 96; mean: 148.1)							
Q1 (< 53)	1120	173	1293	Ref			
%	86.62	13.38	100				
Q2 (53–109)	1079	248	1327	1.875	0.002	1.265	2.780
%	81.31	18.69	100				
Q3 (110–224)	1035	291	1326	2.321	< 0.001	1.560	3.455
%	78.05	21.95	100				
Q4 (> 225)	909	418	1327	5.963	< 0.001	3.885	9.154
%	68.5	31.5	100				

AA, Aberdeen angus; CH, Charolais; FR, Friesian; HE, Hereford; LM, Limousin.

Univariable mixed models, controlling for herd clustering, was fitted to each independent variable respectively and reported. Throughout, linear predictors were categorised based on quartiles. The BVD serodiagnosis result was dichotomised by assuming inconclusive test results were negative, following Barrett et al.^23^. Breed categories were simplified by classing “pure” breeds with their reported “cross breeds”, for example, both Aberdeen angus (AA) and Aberdeen Angus crosses (AAX) were considered being within the same category. Due to their being several rarer breeds that were poorly represented in the dataset, we also classified any breed types with < 700 observations as “other”, yielding a categorical predictor with 6 classes. Movements of animals between enterprises during the last year was simplified, with animals having 3 or more moves being grouped into a single category.

Multivariable models were fitted controlling for the non-independence of observations from the same herd with the inclusion of a random effect. Year was controlled for as a fixed effect. All other potential explanatory variables were added as fixed effects. Backward elimination was used to build parsimonious candidate models using a cut-point of P  < 0.05, but preferred models were informed by information criteria. Both Akaike’s and Bayesian information criteria were used to compare competing models. Bayesian information criteria penalise models with greater numbers of parameters, relative to Akaike’s information criteria. Models were considered equivalent if the difference in AIC was < 2, and models with a difference range of 2–7 having some support^29^. Following Raftery^30^, differences greater than 10 in the BIC value between models was considered very strong evidence against the more complex model. To improve inference and avoid overfitting, bias corrected bootstrapping estimates with 1000 iterations was also calculated^31,32^ for the final model. This approach resamples the distribution, with replacement, and iteratively fits models while collecting the estimated standard errors and associated 95%CI. A bias statistic calculates how the bootstrap estimates deviates from the fitted model, with inference based on the confidence intervals derived from the bootstrapped dataset, providing a better indication of the generalisability (by avoiding overfitting) of the model. 1000 iterations were chosen as we were interested in estimates of the 95%CI, as well as the standard error estimates^33,34^. All analyses were undertaken with Stata 16.1MP^35^.

## Results

There were 5542 IBR test results in the dataset from 3687 last herds, of which 213 were missing contemporaneously sampled BVD test results. Furthermore, there was 56 animals which were over 30-months of age (mean: 48.4 months; IQR: 33–42 months; Max: 223 months). These data were not included in any further modelling (therefore, n = 5273 for all multivariable models), but their descriptive statistics are presented in the Supplementary material. There was no significant difference in the proportion IBR positive in the 213 records with missing data (OR: 1.20; P = 0.252; 95%CI: 0.88–1.65), however the seroprevalence amongst the 56 older animals was significantly higher (35.7%) than the proportion positive amongst the study cohort (21.43%; OR: 2.04; P  = 0.011; 95%CI: 1.17–3.53).

### Univariable analysis

The seroprevalence of IBR was 21.43% (1130/5273; 95%CI: 20.32–22.53), with an additional 61 inconclusive results. Estimated true seroprevalence was 21.41% (95%CI: 20.31–22.55%).

The seroprevalence of BVD from the random sample was 4.67% (246/5273; 95%CI: 4.15–5.30), with an additional 68 suspect (inconclusive) results.

There was a higher proportion of animals BVD seropositive when seropositive for IBR (n = 78; 6.96% positive), in comparison with animals with seronegative IBR results (n = 168; 4.11%; Pearson χ^2^ (1) = 15.80; Pr < 0.001). Univariate mixed effect logit models found significant positive associations between IBR test results and BVD status, with random effects for either the last herd (OR: 1.92; 95%CI: 1.13–3.24) or birth herd (OR: 2.27; P = 0.001; 95%CI: 1.40–3.68). Models accounted for clustering effects within the last-herd cattle were sampled, relative to herds within which they were born, appeared to fit the data better (ΔAIC and ΔBIC > 300). The marginal predicted IBR seroprevalence being 25.44% (95%CI: 18.19–20.17) in animals coinfected with BVD, and 19.40% (95%CI: 18.19–20.62) otherwise.

The relationship between the probability of IBR seropositivity and selected animal- and herd-level factors are presented in Table 1 below. All univariable models were fitted with a random effect for the last-herd the animal was sampled in. The seroprevalence was significantly higher in 2020 (23.32%; 95%CI: 21.23–25.41%; marginal predictions from a random effect model) than in 2018 (17.61%; 95%CI: 16.21–19.00%). At the animal level, IBR risk appeared to peak for animals in second age-quartile at 24.4–26.4 months old. While Aberdeen angus (and their crosses) appeared to have the higher IBR positivity rates, the actual variation across all breed categories was not significant (Wald χ^2^(DF: 5) = 7.40; Prob > χ^2^ = 0.193). Males appeared to be a slightly increased risk, relative to female animals, though the size effect was small. Animals who were sampled at a different herd that where they were born were significantly more likely to test positive for IBR, and furthermore, it appears that there was a linear increase in risk with the more movements made in the last year (see ORs in Table 1). Animals sampled in beef and dairy herd-types appear to be of greater risk than suckler or “other” herd types, though there was less variation in IBR positivity risk depending on the birth herd types. Herd size was a significant positively associated risk factor for IBR positivity, for both birth herd and the herd the animal was sampled in. However, there appeared to be a greater effect size for the last herd the animal was sampled within, relative to the birth herd size effect.

### Multivariable model

Several competing models had similar AIC values, with the largest difference being ΔAIC = 8.17 (Table 2 Supplementary material). Given the difference in the number of parameters between models, the BIC was more discriminatory (max ΔBIC = 120.98), ranking the most parsimonious (DF: 11) model highest. There was strong evidence to suggest that there was significant clustering at the [last] herd level (LR test of ρ = 0: χbar^2^(01) = 464.08; Prob >  = χbar^2^ < 0.001). The final model exhibited an AUC of 0.65 (95%CI 0.63–0.67). Bootstrapping indicated there were some bias in the final model (BIC ranked) predictions, based on the biased-corrected confidence intervals. The final model with observed odds-ratios with bootstrapped standard errors and bias-corrected 95%CI is presented in Table 2.

**Table 2:** Multivariable model of the risk of BoHV1 animal-level test positivity in relation to animal- and herd-level factors.

	Obs. OR	Obs. P-value	Bootstrap	Bootstrap	Normal-based bootstrap		Bias corrected bootstrap	
			SE	P-value	Lower 95%CI	Upper 95%CI	Lower 95%CI	Upper 95%CI
Last herd size (quartiles)								
1	Ref.							
2	1.722	0.001	0.336	0.005	1.175	2.525	1.265	1.907
3	2.239	< 0.001	0.458	< 0.001	1.500	3.343	1.794	2.280
4	4.719	< 0.001	1.029	< 0.001	3.078	7.234	–	–
No. moves								
0	Ref.							
1	2.005	< 0.001	0.383	< 0.001	1.380	2.915	1.267	2.365
2	2.632	< 0.001	0.572	< 0.001	1.718	4.030	1.642	3.164
≥ 3	3.494	< 0.001	0.884	< 0.001	2.128	5.736	2.143	4.463
Sex								
Female	Ref.							
Male	1.231	0.032	0.179	0.154	0.925	1.637	0.975	1.287
Year								
2018	Ref.							
2020	0.634	< 0.001	0.099	0.003	0.467	0.861	0.518	0.939
BVDV status								
Neg.	Ref.							
Pos.	1.602	0.048	0.487	0.121	0.883	2.906	0.881	2.875
Constant	0.038	< 0.001	0.011	< 0.001	0.021	0.067		
Herd level var.	1.754		0.098		1.572	1.958		
ICC (rho)	0.483		0.028		0.429	0.538		

**Figure 1:**
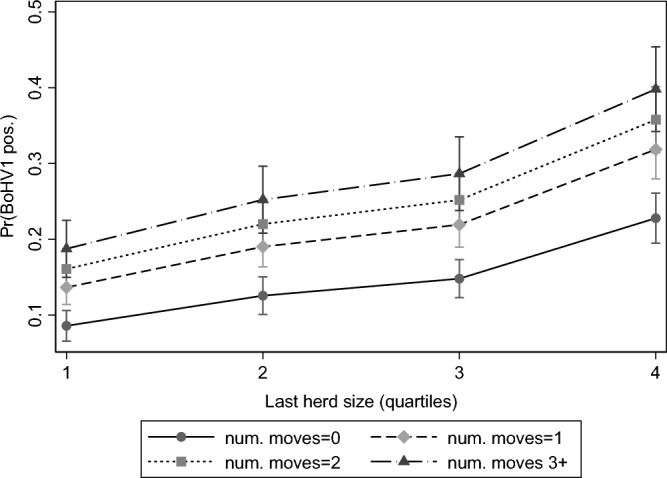
The marginal predicted difference in probability of an animal testing positive to IBR depending on herd size and animal movements, controlling to clustering effects of animals residing in the same herd at test.

BoHV1 positivity was associated with increasing herd size, with animals sampled from herds within the largest quartile having 4.719 times the odds of being seropositive relative to animals coming from herds with sizes within the first quartile (Fig. 1). The probability of an animal being BoHV1 positive was also significantly associated with the number of movements that the animal had during the previous year prior to sampling, with animals with ≥ 3 movements being 3.494 (bias corrected 95%CI: 2.143–4.463) times the odds of test positivity relative to animals that did not make any movements (Fig. 1). Year was retained in the final model, with lower animal-level risk of test positivity in 2020 relative to 2018 (OR: 0.634; bias corrected 95%CI: 0.518–0.939). Point estimates of the odds ratios for BVDV status and animal sex, suggested higher risk of BoHV1 positivity when animals were males (OR: 1.231) and BVDV positive (OR: 1.602), respectively. However, bias corrected 95%CI of the odds ratios straddled 1 for both being male (bias corrected 95%CI: 0.975–1.287) and BVDV status (bias corrected 95%CI: 0.881–2.875), suggesting insufficient statistical support that these associations could be considered robust (Table 2).

## Discussion

Over the two years of the study, we found an average BoHV-1 seroprevalence of 21.43%. However, there was a significant reduction in the seroprevalence from 23.32% in 2018 to 17.61% in 2020. The current study differs from previous studies on BoHV-1 in Ireland in that it focuses on cattle less than 30 months of age in commercial cattle herds (see Table 3 for an overview of previous studies). In a study of 161 suckler cow herds, Barrett et al. (2018) found an overall mean within-herd prevalence of 39.8% and a herd-level prevalence of 90%^8^. In a study of young bulls being submitted to AI (artificial insemination), O’Grady et al.^15^ found an animal seroprevalence of 28% and a herd level prevalence of 73%. A previous study examining the seroprevalence of BoHV-1 among cattle tested for the national brucellosis eradication recorded a herd-level seroprevalence of 74.9%^11^. While we do not have a herd level prevalence from this study, the adult animal level prevalence tended to be lower than those previously reported in Ireland (cf: Ring et al.^36^). The result from the present study may, in part, be related to the age of the cattle surveyed in this study, as other studies have focused on older age cohorts. Indeed, Martinez-Ibeas et al.^12^ found that increasing age was a significant risk factor for BoHv-1 seropositivity. An exception that proves the rule, is the study by Sayers et al.^7^. Sayers et al.^7^ sampled 2171 weanling with a mean age of only 291 days and found a very low exposure with 5.4% of these calves being seropositive for BoHV1.

**Table 3:** Review of previous studies relating to apparent prevalence of BoHV1 exposure in cattle in Ireland.

Study	Year	Herd, within-herd, or animal level	Serum or bulk milk	n	Sample year(s)	Apparent Prevalence
^15^	2008	Herd	Serum	41 beef herds [bulls]	2007	73% (30/41)
^15^	2008	Within-herd	Serum	30 [infected] beef herds [bulls]	2007	28% (SD: 20); Median herd size: 55
^11^	2011	Herd	Serum	1175 dairy and beef herds	2009	75% (95% CI 70–80%)
^11^	2011	Herd	Bulk milk	111 dairy herds	2009	71% (79/111)
^7^	2015	Herd	Bulk milk	305 dairy herds	2009	80% (244/305)
^7^	2015	Animal	Serum	2171 weanlings	2009	5% (117/2171)
^12^	2015	Animal	Serum	529 bulls	2009	17% (87/529)
^36^	2018	Animal	Serum	6534 female cattle (24 herds)	2010–2013	26%
^36^	2018	Animal	Serum	10,669 cows (67 herds)	2015	23%
^8^	2018	Herd	Serum	161 beef herds	2014–2015	90%
^8^	2018	Within-herd	Serum	6049 cows	2014–2015	40%

The size of the herd the animal resided in immediately prior to slaughter, the number of herds the animal resided in, the year the animal was sampled in, the sex of the animal and the animal’s BVD serostatus were all significant in the final logistic regression model. Herd size is a well-recognised risk factor associated with the occurrence of several diseases in Ireland and elsewhere including bovine tuberculosis^37^, BVD^23^ and BoHV-1^5,11^. Increasing herd size increases the probability of an individual animal becoming exposed to pathogens when other infected cattle are present in that herd. This may be especially important in the epidemiology of BoHV-1, where infection may be re-activated in latently infected cattle when they become stressed^38^. Cattle slaughtered out of herds with more than 225 cattle, were almost 5 times as likely to seroconvert to BHV-1 as cattle slaughtered out of herds with less than 53 cattle. This finding would suggest that there could be value in focusing an BoHV-1 control programme in larger herds, and a strategy that could be tested using simulation tools (for example, Brock et al.^39^).

The number of herds the animal resided in over its lifetime was found to be the next most important risk factor, which has been reported previously^40^. The risk of serconversion among cattle that resided in two herds was twice that of those that resided in only one herd from birth. This increased to 2.6 times and 3.5 times to cattle that resided in three and four or more herds respectively. Similar to the herd size risk factor, movement between herds represents an opportunity for increased exposure to infected cattle, but also could represent a stressor^41^ that could impact on susceptibility or reactivation. Another aspect of movement not addressed in this study was the movement of cattle was facilitated through cattle marts, where several hundred cattle may be assembled from several herds on the same day. Marts can be important connecting nodes amongst cattle movement networks^42^, which can facilitate infectious pathogen spread^43^. This potential exposure at markets could provide an added opportunity for the transmission and spread, across connecting trading ‘nodes’ of the network, of BoHV-1.

We found male cattle were 1.2 times more likely to seroconvert to BoHV-1 than their female counterparts, however, bootstrapping of the final multivariable model suggested that this association was not robust. There is no obvious biological reason for a sex bias, but the pattern has been reported elsewhere in the literature (e.g.^5,44^). However, such sex biases in terms of risk may relate to the overall management of the males versus females. We did not make a distinction between bulls and bullocks (steers) and we would suggest that bulls may be more intensively managed than either bullocks or heifers and this may have contributed to any differential risk, but we do not have the data to investigate this further. Bulls may also be exposed to more at-risk contacts, either by their behaviour at the individual-level or by their inter-herd movements^44^.

We found that there were an increased odds of cattle slaughtered in 2018 being test positive relative to animals slaughtered in 2020, controlling for covariables within the model. In addition, we found that cattle seropositive for BVD were 1.6 times more likely to seroconvert to BoHV-1 than cattle seronegative for BVD, controlling for year. While these were both significant independent variables in the final model, they may be associated. There has been a BVD eradication programme in Ireland since 2013, where the herd level prevalence of BVD has decreased from 11% in 2013 to 0.59% in 2020^45^. The variance explained by the “year” variable is possibly reflecting some of the effects of BVD control, the biosecurity efforts of farmers in Ireland in recent years^46^, and the unobserved dynamics not captured by this analysis.

A previous Irish study of 6000 beef suckler cows documented an association between the presence of BVDV antibody positive animals and seroconversion to BoHV-1^8^, which is possibly due to the immunosuppressive effects of BVD. This would suggest that the eradication of BVD may assist in reducing the seroprevalence of BoHV-1. A study by Sayers et al.^7^ found that 72% of herds sampled for BVD and BoHV-1 using bulk milk samples were concurrently infected in Ireland in 2009. In fact, during that study, only 10 herds (from 305 total herds) were bulk milk seronegative to both pathogens. Even though there was evidence of coinfection associations in previous studies with BVDV in Ireland (e.g.^7,8^), in the present study, this association was not very statistically robust. Despite this, we know that biosecurity and other infectious disease control interventions from one programme, can have indirect benefits to other pathogens without formal control programmes.

### Limitations

The data generated for this study was part of overall surveillance activities and not solely for the surveillance of BoHV-1. The serosurvey was not carried out in 2019, so data from the middle year of the study period is not available. The study was observational and retrospective so therefore any associations cannot be necessarily considered causative. However, most of these associations were previously documented and are generally biologically plausible. We are confident that vaccination would not have interfered with the outcome, as the test kit used in the study for BoHV-1 Ab testing is based in gE detection, a non-structural protein deleted in all commercially available vaccines in Ireland, thus differentiating natural infection from vaccinal antibodies.

## Conclusions and implications

This study has demonstrated the BoHV 1 status of cattle at slaughter is associated with the size of the herd the cattle are slaughtered out of, the number of herds the animal resided within and, to a lesser degree, the cattle’s BVD serostatus. This emphasises the need to focus on cattle in larger herds and cattle which move between multiple herd in any effort to control BoHV-1, a finding that could be useful for several other countries currently working towards eradication^47^. This study also emphasises the potential benefits the eradication of BVD will bring to the control of BoHV-1.

### Supplementary Information

Supplementary Tables.

## Data Availability

The datasets analysed during the current study are available from the data controller DAFM on reasonable request and subject to anonymisation of any personal data compliant with GDPR. For data requests please contact the first author or ohssu@agriculture.gov.ie.
