# Supplementary material for: Bovine Herpes Virus Type 1 (BoHV-1) seroprevalence, risk factor and Bovine Viral Diarrhoea (BVD) co-infection analysis from Ireland
Source: Sci Rep. 2024 Jan 9;14:867. doi: 10.1038/s41598-023-50433-5 (PMC10776861; doi:10.1038/s41598-023-50433-5)
Supplement: Supplementary file 1 — Supplementary Tables. [file 41598_2023_50433_MOESM1_ESM.docx]

# Supplementary material

There were 213 observations with missing BVD test results. A tabulation of these IBR results are presented below. Note the very similar proportions positive for IBR.

Table S1: Two by two table of the proportion of IBR test results with missing BVD test results.

|  | Missing |  |  |
| --- | --- | --- | --- |
| ibr_bin | 0 | 1 | Total |
|  |  |  |  |
| 0 | 4,180 | 160 | 4,340 |
|  | 96.31 | 3.69 | 100 |
|  |  |  |  |
| 1 | 1,150 | 53 | 1,203 |
|  | 95.59 | 4.41 | 100 |
|  |  |  |  |
| Total | 5,330 | 213 | 5,543 |
|  | 96.16 | 3.84 | 100 |

Of the animals with full test and covariate records, 56 were older than 30 months. Note the higher proportion positive (35.7%) in the older cohort relative to the rest of the sampled population (21.43%).

Table S2: The proportion of animals >30 months old that were positive for IBR.

|  |  | >30mths |  |
| --- | --- | --- | --- |
| ibr_bin | 0 | 1 | Total |
|  |  |  |  |
| 0 | 4,143 | 36 | 4,179 |
|  | 78.57 | 64.29 | 78.42 |
|  |  |  |  |
| 1 | 1,130 | 20 | 1,150 |
|  | 21.43 | 35.71 | 21.58 |
|  |  |  |  |
| Total | 5,273 | 56 | 5,329 |
|  | 100 | 100 | 100 |

Table S3: Comparison of candidate multivariable models of BoHV1 seropositivity risk in terms of AIC and BIC metrics. Note, ΔAIC and ΔBIC represent the difference between the highest rank model and the current ranked model; models with ΔAIC<=7 or models with ΔBIC<=10 indicate similar fitting models to the data.

| Independent variables | N | ll(model) | df | AIC | BIC | BIC rank | AIC rank | ΔAIC | ΔBIC |
| --- | --- | --- | --- | --- | --- | --- | --- | --- | --- |
| Lastsize, num_moves, sex, sample_yr, bvd | 5,273 | -2393.58 | 11 | 4809.17 | 4881.44 | 1 | 10 | 5.46 | 0.00 |
| Lastsize, num_moves, sex, herd_type_last, sample_yr, bvd | 5,273 | -2389.76 | 14 | 4807.51 | 4899.50 | 2 | 7 | 3.80 | 18.06 |
| Lastsize, num_moves, sex, age, sample_yr, bvd | 5,273 | -2389.78 | 14 | 4807.56 | 4899.55 | 3 | 8 | 3.86 | 18.11 |
| Lastsize, num_moves, sex, breed_simpl, sample_yr, bvd | 5,273 | -2388.15 | 16 | 4808.30 | 4913.43 | 4 | 9 | 4.59 | 31.99 |
| Lastsize, num_moves, sex, age, herd_type_last, sample_yr, bvd | 5,273 | -2386.11 | 17 | 4806.22 | 4917.91 | 5 | 3 | 2.51 | 36.47 |
| Lastsize, num_moves sex, breed_simpl age, sample_yr, bvd | 5,273 | -2384.26 | 19 | 4806.52 | 4931.35 | 6 | 4 | 2.81 | 49.91 |
| Lastsize, num_moves, sex, breed_simpl age, herd_type_last sample_yr, bvd | 5,273 | -2380.52 | 22 | 4805.03 | 4949.58 | 7 | 2 | 1.33 | 68.14 |
| Lastsize, num_moves, sex, ever_move breed_simpl, age, herd_type_last, sample_yr, bvd | 5,273 | -2378.85 | 23 | 4803.71 | 4954.82 | 8 | 1 | 0.00 | 73.38 |
| lastsize num_moves sex ever_move, breed_simpl age herd_type_last, herd_type_birth sample_yr bvd, | 5,273 | -2377.63 | 26 | 4807.27 | 4978.10 | 9 | 6 | 3.56 | 96.66 |
| lastsize birthsize herd_type_birth herd_type_last, num_moves, ever_move sex, breed_simpl, age, sample_yr bvd | 5,273 | -2376.94 | 29 | 4811.88 | 5002.42 | 10 | 11 | 8.17 | 120.98 |
| Lastsize, num_moves, sex, sample_yr, bvd, county | 5,273 | -2367.58 | 36 | 4807.17 | 5043.70 | 11 | 5 | 3.46 | 162.26 |
